# A Review of Anti-Inflammatory Compounds from Marine Fungi, 2000–2018

**DOI:** 10.3390/md17110636

**Published:** 2019-11-09

**Authors:** Jianzhou Xu, Mengqi Yi, Lijian Ding, Shan He

**Affiliations:** Li Dak Sum Yip Yio Chin Kenneth Li Marine Biopharmaceutical Research Center, College of Food and Pharmaceutical Sciences, Ningbo University, Ningbo 315832, China; 176001796@nbu.edu.cn (J.X.); ymqnbu@163.com (M.Y.)

**Keywords:** marine-derived fungi, marine natural products, anti-inflammatory

## Abstract

Inflammation is a generalized, nonspecific, and beneficial host response of foreign challenge or tissue injury. However, prolonged inflammation is undesirable. It will cause loss function of involve organs, such as heat, pain redness, and swelling. Marine natural products have gained more and more attention due to their unique mechanism of anti-inflammatory action, and have considered a hotspot for anti-inflammatory drug development. Marine-derived fungi are promising sources of structurally unprecedented bioactive natural products. So far, a plethora of new secondary metabolites with anti-inflammatory activities from marine-derived fungi had been widely reported. This review covers 133 fungal metabolites described in the period of 2000 to 2018, including the structures and origins of these secondary metabolites.

## 1. Introduction

Inflammation has been described as the general, complex, and beneficial immune system in response to external challenges or tissue damage [1]. It can ultimately restore tissue structure and function. Without inflammation, wounds would never be healed. However, if inflammation is not controlled for a long time, genetic mutations caused by immune cell-derived reactive oxygen species and numerous pathogenesis involved in the inflammatory response might contribute to many diseases, for example, cancer, multiple sclerosis, atherosclerosis, arthritis, heart disease, insulin resistance, and others [2,3]. At the same time, it will cause excessive expression of various inflammatory media to produce conditions conducive to many chronic diseases occurrence such as cancer, neurodegenerative disorders, diabetes, and cardiovascular diseases [4,5].

During the inflammatory process, the stimulated immune monocytes and macrophages trigger the transactivation of several important transcription factors. The well-known inflammatory signal pathway is NF-*κ*B signal pathway called a canonical pathway [6]. NF-*κ*B located in the cytoplasm is composed of two subunits (p50 and p65) as an inactive heterodimer bond to I*κ*B-*α,* which is an inhibitory protein. In the stimulated condition, the phosphorylation and proteolytically degradation of I*κ*B-*α* allows translocation of NF-*κ*B into nuclear to regulate target gene transcription by binding to the *κ*B site in the DNA’s structure [7]. The NF-*κ*B transactivation will increase activities of the downstream responses such as pro-inflammatory cytokines (such as IL-1*β* IL-6, and TNF-*α*) [8], the important pro-inflammatory enzymes (such as iNOS and COX-2) and their derived production NO and PGE_2_, respectively [9]. In addition to NF-*κ*B activation, another important pathway, MAPK signal pathway such as extracellular signal-regulated kinases (ERK), p38 MAPK, and cJun NH_2_-terminal kinases (JNK) [7], also can be activated by inflammation and regulates the transcription of various inflammatory-related genes then overexpress the downstream inflammatory response [10]. Amounts of inflammatory mediators and factors are involved in cell damage and inflammatory such as redness, pain, fever, and swelling [11,12]. Therefore, inhibition of the overproduction of these is an important target in the treatment of inflammatory disease [13]. Researchers usually evaluated the anti-inflammatory activity by the suppressed expression of pro-inflammatory cytokines, the pro-inflammatory enzyme of COX-2, iNOS and their derived production, and the various inflammatory-related protein in NF-*κ*B and MAPK signal pathways in immune monocytes and macrophages (BV2 cells, RAW264.7 cells and more) stimulated by LPS in vitro [14], or by the inhibited swelling rate in mouse ear edema model induced by phorbol myristate acetate (PMA) in vivo [15].

Toward the aim of discovering new natural products with anti-inflammatory activities, researchers spend a lot of time and energy to discover novel sources in different environment. The oceans with their unique aquatic environment and plentiful biodiversity has drawn attention for the rich source of diverse secondary metabolites with significantly anti-inflammatory, antitumor, antimicrobial, antiviral, antimalarial, and anti-oxidant activities [16,17]. According to the MarinLit database (http://pubs.rsc.org/marinlit), annually more than 1200 novel natural products are reported from a variety of marine sources, such as algae, ascidians, bryozoan, corals, microorganisms, sea hares, sea squirts, sponges, and so on [18,19]. Since Alexander Fleming discovered penicillin in 1928 from *Penicillium* [20], people have never stopped discovering new drugs from fungi. Fungi is a crucial source as lead structures for novel pharmaceuticals [21]. Fungi also act as an important ecological role in the marine environment, such as pathogens of marine invertebrates, primary decomposers, and obligate symbionts [22]. Especially, marine-derived fungi play a vital role in the discovery of new anti-inflammatory drugs. Many novel secondary metabolites showing potent anti-inflammatory activities have been discovered from fungi residing in or on algae, sediments, water, and corals. Due to its unique mechanism of action, marine fungal compounds have received more and more attention and become one of the hotspot area for the development of anti-inflammatory drugs.

**Figure 1:**
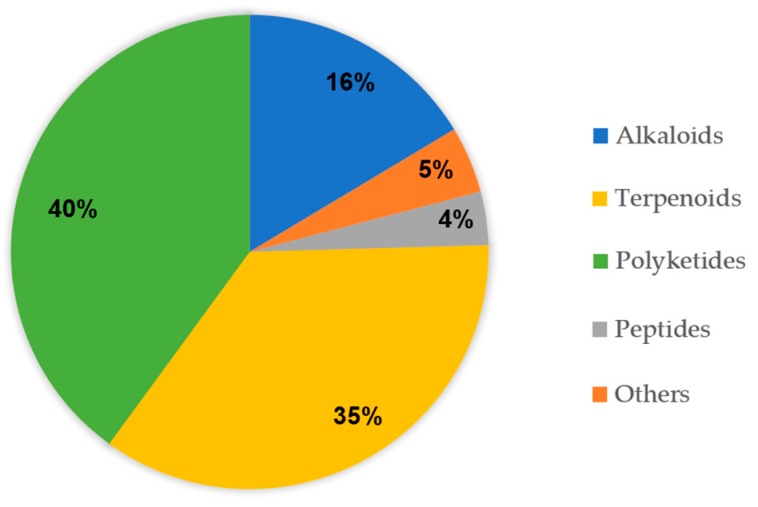
Anti-inflammatory compounds isolated from marine fungi according to structure types.

**Figure 2:**
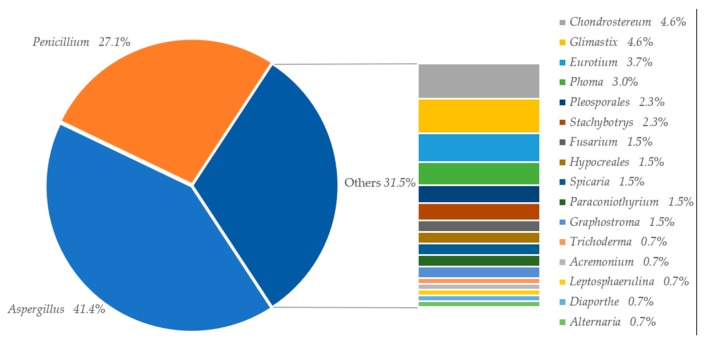
The sources of marine fungal compounds with anti-inflammatory activities.

This review provides a comprehensive overview of 133 marine fungi-derived anti-inflammatory compounds assorted into five structure types, including alkaloids (Table 1), terpenoids (Table 2), polyketides (Table 3), peptides (Table 4), and others (Table 5), which show the proportion of structure types, 16%, 35%, 40%, 5%, and 4%, respectively (Figure 1). A large proportion of the secondary metabolites produced by *Aspergillus* (41.4%), and *Penicillium* (27.1%; Figure 2). Some of these natural products, such as preussin G (5) and preussin I (7), were shown to have remarkable anti-inflammatory activities even stronger than these of the positive control [23]. Therefore, these compounds will emerge as new lead structures for potential anti-inflammatory drugs.

## 2. Alkaloids

**Figure 3:**
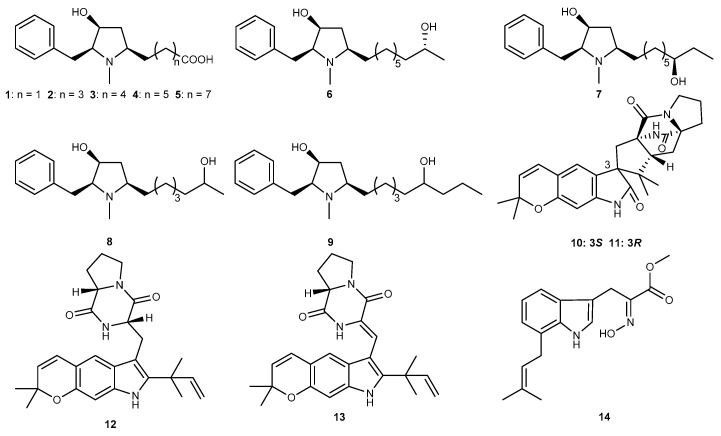
Chemical structures of compounds 1–14.

The fungus *Aspergillus flocculosus* 16D-1 was associated with the inner tissue of the sponge *Phakellia fusca* colonizing in Yongxing Island, China, and produced new pyrrolidine alkaloids, preussins C–I (1–7, Figure 3) and (11*R*)/(11*S*)–preussins J and K (8 and 9, Figure 3) [23]. Compounds 5 and 7 showed remarkable anti-inflammatory activity toward interleukin (IL)–6 production in lipopolysaccharide (LPS)–activated THP-1 cells with IC_50_ values of 0.11 μM and 0.19 μM, which was stronger than that of corylifol A, a positive control with the IC_50_ value of 0.67 μM, while other compounds possessed moderate inhibitory effects, with IC_50_ values in the range of 2.3 to 22 μM [23]. Chemical examination of the cultured mycelium of the fungus *Aspergillus versicolor* collected from the mud of the South China Sea led to the isolation of some novel linearly fused prenylated indole alkaloids: asperversiamides B, C, F, and G (10–13, Figure 3). These compounds exerted potential inducible nitric oxide synthase (iNOS) inhibitory effects and suppressed the release of nitric oxide (NO) in LPS-induced RAW264.7 cells. And of these compounds, asperversiamide G showed a potent inhibitory effect against iNOS with the IC_50_ value of 5.39 μM, while others exhibited weak activities with IC_50_ values ranging from 9.95 to 16.58 μM. Considering the significant inhibitory activity of asperversiamide B, it can synthesize the potential derivatives in the development of new anti-inflammatory drugs for the treatment of various related disorders [24]. A prenylated tryptophan derivative, luteoride E (14, Figure 3) was purified from the coral-associated fungus *Aspergillus terreus* associated with the coral *Sarcophyton subviride*, which was gathered from the coast of Xisha Island in the South China Sea. This compound exhibited inhibitory activity against NO production with IC_50_ value of 24.65 μM in LPS-stimulated RAW264.7 cells [25].

**Figure 4:**
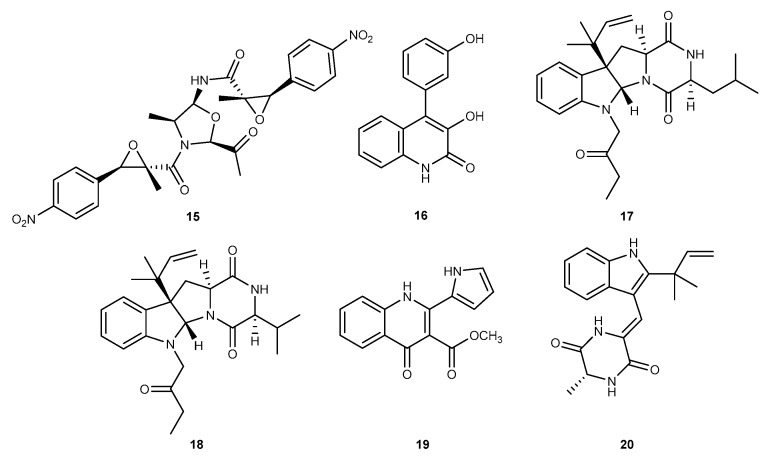
Chemical structures of compounds 15–20.

Chrysamide C (15, Figure 4), a new dimeric nitrophenyl *trans*-epoxyamides, was obtained from the marine-derived fungus *Penicillium chrysogenum* SCSIO41001, collected from deep sea sediment in the Indian Ocean [26]. Chrysamide C was observed to be most active on inhibitory activity toward the proinflammatory cytokine IL-17 production, while inhibitory rate of chrysamide C was found to 40.06% at 1.0 μM [26]. A new quinolone alkaloid, viridicatol (16, Figure 4), was discovered in the marine-derived fungus *Penicillium* sp. SF-5295 [27]. Compound 16 displayed anti-inflammatory potency in LPS-stimulated RAW264.7 cells and BV2 cells. Viridicatol inhibited the production of iNOS-derived NO in RAW264.7 cells with IC_50_ values of 46.03 μM in RAW264.7 cells and 43.03 μM in BV2 cells and suppressed the production of cyclooxygenase-2 (COX-2)-derived prostaglandin E_2_ (PGE_2_) with an IC_50_ value of 30.37 μM in RAW264.7 cells and 34.20 μM in BV2 cells. Compound 16 also inhibited the mRNA expression of IL-1*β*, IL-6, and tumor necrosis factor-*α* (TNF-*α*), which were pro-inflammatory cytokines [27]. In the further evaluation, compound 16 exerted anti-inflammatory activity through suppressing the NF-*κ*B pathway by blocking the phosphorylation of inhibitor kappa B (I*κ*B)-α, and suppressing the translocation of NF-*κ*B dimers, namely p50 and p65 in RAW264.7 macrophages and BV2 microglia induced by LPS [27]. Another study on the *Penicillium* sp. derived from a deep ocean sediment resulted in the discovery of two novel diketopiperazine alkaloids, brevicompanines E and H (17 and 18, Figure 4) [28]. These compounds were shown to have the moderate anti-inflammatory activity to inhibit NO production in LPS-induced BV2 microglial cells, with IC_50_ values of 27 and 45 μg/mL, respectively [28]. In addition, these compounds displayed no cytotoxic effect at these concentrations. Some evidence indicate that substituents at the *N*-6 position were significant for inhibitory activity of NO production [28]. These compounds may be a potential for finding a chemotherapeutic candidate that has anti-inflammatory with no cytotoxic effects [28]. A soft coral samples collected at Terra Nova bay, Antaratica, resulted in the isolation of *Penicillium* sp. SF-5995, which led to the isolation of a pyrrolyl 4-quinoline alkaloid, methylpenicinoline (19, Figure 4) [29]. Compound 19 suppressed the NO and PGE_2_ production by attenuating iNOS and COX-2 expression, respectively, in LPS-stimulated RAW264.7 macrophages and BV2 microglia with the IC_50_ values of ranging from 34–49 μM [29]. Furthermore, compound 19 inhibited the pro-inflammatory cytokine IL-1*β* production [29]. In the further study, compound 19 suppressed the expression of pro-inflammatory cytokines through the NF-*κ*B and mitogen-activated protein kinase (MAPK) pathway in LPS-induced RAW264.7 macrophages and BV2 cells [29]. Another marine fungus *Eurotium* sp. SF-5989 was also isolated from a soft coral collected at Terra Nova bay, Antarctica. Chemical investigation of the fungus *Eurotium* sp. SF-5989 afforded a diketopiperazine-type indole alkaloid, neoechinulin A (20, Figure 4) [30]. Compound 20 suppressed the production of pro-inflammatory mediators, NO and PGE_2_, and these inhibitory activities were mediated by inhibiting the expression of COX-2 and iNOS in RAW264.7 macrophages stimulated by LPS. The anti-inflammatory mechanism of compound 20 was due to attenuation of two major signaling pathways, NF-*κ*B pathway and MAPK signaling pathway in LPS-stimulated RAW264.7 macrophages and BV2 microglia [30].

## 3. Terpenoids

**Figure 5:**
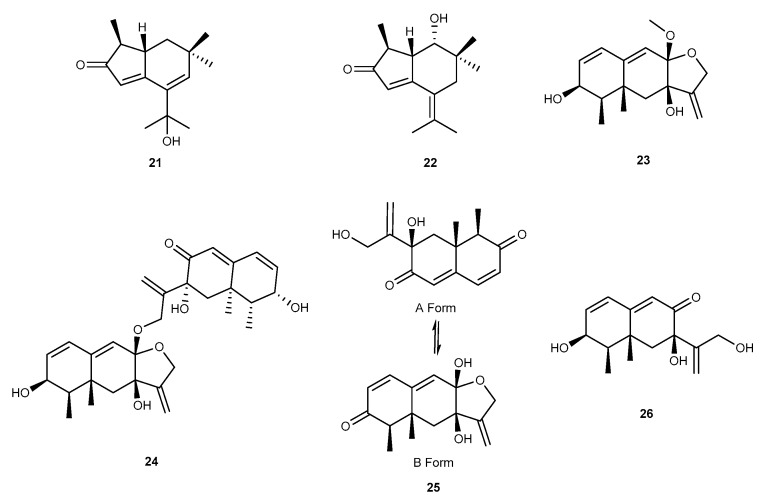
Chemical structures of compounds 21–26.

Two novel brasilane sesquiterpenoids, brasilanones A and E (21, 22, Figure 5), were separated from the extract of the marine-derived fungus *A. terreus* CFCC 81836, which displayed moderate inhibitory activities against NO production with inhibition rates of 47.7% and 37.3% at 40 μM in RAW264.7 mouse macrophages induced by LPS [31]. Liyan Wang et al. firstly reported three new eremophilane-type sesquiterpenoids of dihydrobipolaroxin B–D (23–25, Figure 5) and a known sesquiterpene of dihydrobipolaroxin (26, Figure 5). These compounds were isolated from a deep sea-derived fungus, *Aspergillus* sp. SCSIOW2, from a deep marine sediment sample gathered from the South China Sea at a depth of 2439 m [32]. All of these compounds were shown to have moderate anti-inflammatory effects to inhibit NO induced by LPS/INF-*γ*. Meanwhile, all four compounds exhibited no cytotoxic effects [32].

**Figure 6:**
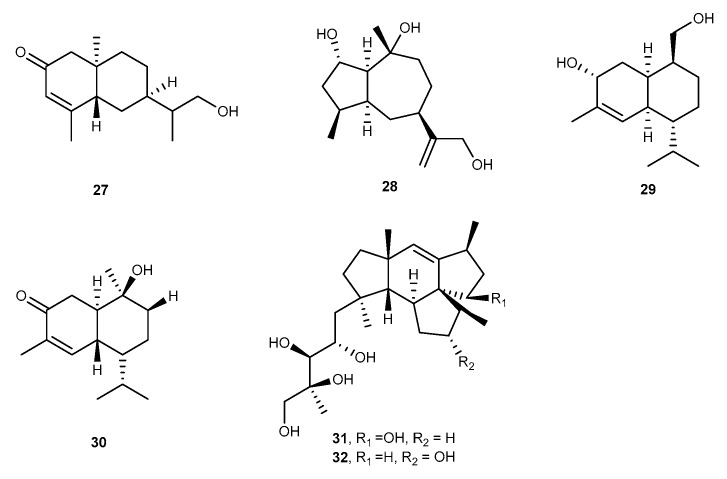
Chemical structures of compounds 27–32.

Thomimarine E (27, Figure 6) was a new eudesmane-type sesquiterpene that was obtained from marine fungus *Penicillium thomii* KMM 4667 [33]. Thomimarine E (27) exhibited anti-inflammatory effect and inhibited the production of NO in LPS-stimulated RAW264.7 cells with inhibition rate of 22.5% ± 5.1% at the concentration of 10.0 μM [33]. *Graphostroma* sp. MCCC 3A00421 isolated from Atlantic Ocean hydrothermal sulfide deposit at a depth of 2721 m produced a new guaiane, graphostromane F (28, Figure 6) [34]. Graphostromane F (28) exhibited considerable inhibitory activity by inhibiting the release of NO in RAW264.7 macrophages induced by LPS with an IC_50_ value of 14.2 μM, which was even lower than the aminoguanidine as positive control with an IC_50_ value of 23.4 μM [34]. Another study on the same *Graphostroma* sp. MCCC 3A00421 resulted in the discovery of a novel fungal sesquiterpene, khusinol B (29, Figure 6) [35]. Khusinol B (29) was found considerable anti-inflammatory activity in LPS-induced RAW264.7 cells against NO production with IC_50_ value of 17 μM, which was even stronger than that of the positive control with the IC_50_ value was 23 μM [35]. Chemical study of the sea-derived fungus *Hypocreales* sp. strain HLS-104, which was isolated from a sponge *Gelliodes carnosa* colonizing in the South China Sea afforded a derivative, 1*R*,6*R*,7*R*,10*S*-10-hydroxy-4(5)-cadinen-3-one (30, Figure 6) with moderate anti-inflammatory activity [36]. The average maximum inhibition (E_max_) values of this molecule against the production of the NO in LPS-treated RAW264.7 cells was 10.22% at the concentration of 1 μM [36]. William Fenical et al., isolated mangicols A and B (31 and 32, Figure 6) from a marine fungus, *Fusarium heterosporum* CNC-477, which was separated from a driftwood sample collected from Sweetings Cay mangrove habitat, Bahamas [37]. Mangicols A and B were novel sesterterpene polyols that exhibited considerable anti-inflammatory effects in the phorbol myristate acetate (PMA)-induced mouse ear edema assay with the reduction of 81% and 57%, respectively, at the standard of 50 μg per ear which were similar to those of indomethacin, the positive control, with the reduction of 71% [37].

**Figure 7:**
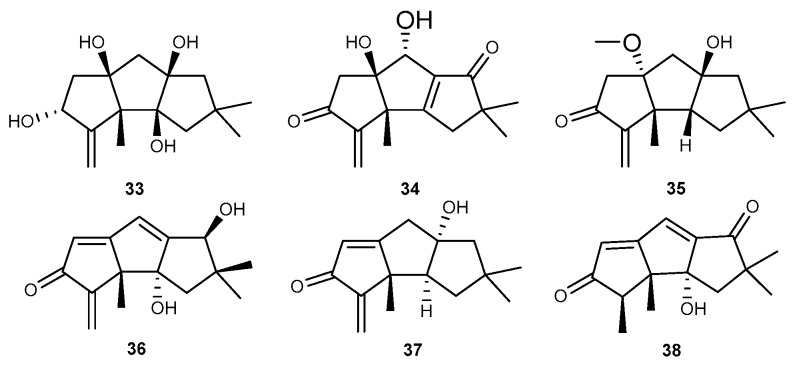
Chemical structures of compounds 33–38.

George Hsiao et al. reported the isolation of eight novel hirsutane-type sesquiterpenoids along with seven known derivatives from the EtOAc extract of the fermented broth of *Chondrostereum* sp. NTOU4196, a fungal strain isolated from the marine red alga *Pterocladiella capillacea*, collected from the northeast and north intertidal zone of Taiwan [38]. Among them, chondroterpenes A, B, H (33–35, Figure 7) and hirsutanol A (36, Figure 7), chondrosterins A and B (37 and 38, Figure 7) showed strong anti-inflammatory effects and possessed the expression of NO in murine BV-2 microglial cells stimulated by LPS at a concentration of 20 μM [38].

**Figure 8:**
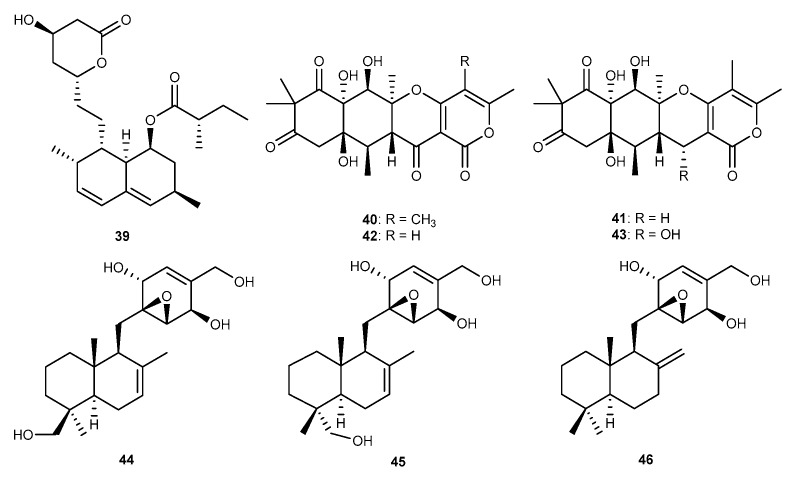
Chemical structures of compounds 39−46.

Lovastatin (39, Figure 8) was purified from the coral-associated fungus *A. terreus* associated with the coral *S. subviride*, collected from the coast of Xisha Island in the South China Sea [25]. This compound showed inhibitory activity on the NO production with IC_50_ value of 17.45 μM in RAW264.7 cells stimulated by LPS [25]. A sea green algal species *Enteromorpha* collected in Dongshi salt pan, Fujian Province, China, resulted in the isolation of a fungus *Aspergillus* sp. ZL0-1b14 [39]. The fungus extracts displaying anti-inflammatory activities was chemically analyzed, which led to the isolation of a family group of new triketide-sesquiterpenoid meroterpenoids, aspertetranones A−D (40−43, Figure 8) [39]. Aspertetranones A−D showed different anti-inflammatory activities. Notably, aspertetranones A and D exhibited the suppress potency against the production of IL-6 in LPS-stimulated RAW264.7 macrophages with 43% and 69% inhibition at 40 μM [39]. Chemical investigation of a marine-derived fungus, *Pleosporales* sp. strain derived from a marine alga *Enteromorpha clathrate* collected from the South China Sea in Hainan Province, yielded three new compounds, pleosporallins A−C (44−46, Figure 8) [40]. They possessed moderate inhibitory activities against the production of proinflammatory cytokine IL-6 in LPS-stimulated RAW264.7 macrophages cells with the inhibition rate about 30.0% compared to control at the concentration of 5−20 μg/mL [40].

**Figure 9:**
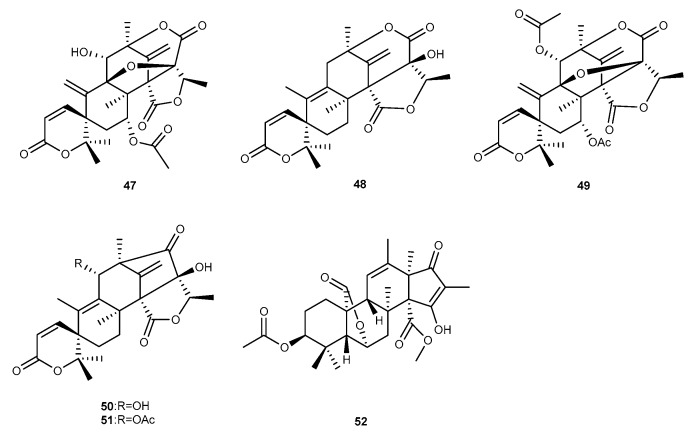
Chemical structures of compounds 47−52.

Jin-Soo Park et al. separated two novel meroterpenoid-type metabolites along with eight known analogs from the ethyl acetate extract of a marine-derived fungal strain *Penicillium* sp. SF-5497, which was isolated from a sample of sea sand collected from Gijiang-gun, Busan [41]. All the isolated metabolites were evaluated for anti-inflammatory activities against NO production in microglial BV-2 cells challenged by LPS, only 7-acetoxydehydroaustinol (47, Figure 9), and four other known analogs austinolide (48, Figure 9), 7-acetoxydehydroaustin (49, Figure 9), 11-hydroxyisoaustinone (50, Figure 9), and 11-acetoxyisoaustinone (51, Figure 9), were shown to have weak inhibitory effects with IC_50_ values of 61.0, 30.1, 58.3, 37.6, and 40.2 μM, respectively [41]. The marine fungus *Penicillium atrovenetum* was shown to produce an undescribed meroterpenoid, citreohybridonol (52, Figure 9) [42]. This compound was found to have anti-neuroinflammatory activity [42].

**Figure 10:**
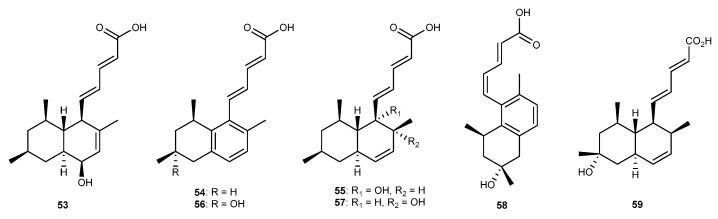
Chemical structures of compounds 53−59.

A new tanzawaic acid derivative, tanzawaic acid Q (53, Figure 10), together with four known analogues, tanzawaic acids A (54, Figure 10), C (55, Figure 10), D (56, Figure 10), and K (57, Figure 10), have been isolated from a marine-derived fungus, *Penicillium steckii* 108YD142, residing in a marine sponge sample collected at Wangdolcho, in the Republic of Korea’s Eastern reef [43]. These compounds considerably inhibited LPS-stimulated NO production in RAW264.7 macrophages cells. Moreover, tanzawaic acid Q reduced the expression of pro-inflammatory mediators such as COX-2 and iNOS and also possessed the production of PGE_2_, TNF-*α,* and IL-1*β* mRNA protein [43]. Marine-derived fungus *Penicillium* sp. SF-6013 derived from the sea urchin *Brisaster latifrons* collected from the Sea of Okhotsk, was shown to produce a new tanzawaic acid derivative, 2*E*,4*Z*-tanzawaic acid D (58, Figure 10), along with two known analogues, tanzawaic acids A (54) and E (59, Figure 10). These three tanzawaic acids inhibited the overproduction of NO in BV-2 microglial cells activated by LPS with IC_50_ values of 37.8, 7.1, and 42.5 μM, respectively [44]. Furthermore, tanzawaic acid A also inhibited the NO production and reduced the expression of iNOS and COX-2 in RAW264.7 and BV2 cells stimulated by LPS [44].

**Figure 11:**
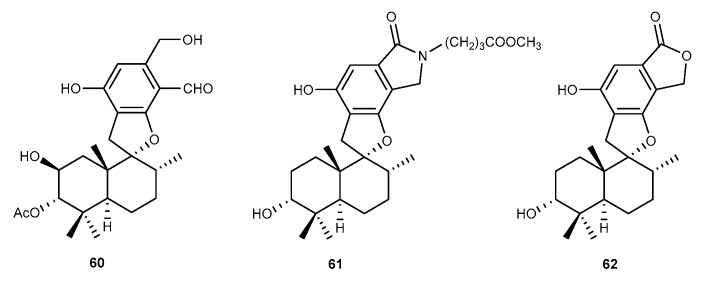
Chemical structures of compounds 60−62.

Three meroterpenoids, named as stachybotrysin C (60, Figure 11), stachybonoid F (61, Figure 11), and stachybotylactone (62, Figure 11) were obtained from *Stachybotrys chartarum* 952 isolated from a marine crinoid (*Himerometra magnipinna*) [45]. Compounds 60, 61, and 62 moderately suppressed the production of NO (the pro-inflammatory mediator) with IC_50_ values of 27.2, 52.5, and 17.9 μM in RAW264.7 macrophages stimulated by LPS [45].

## 4. Polyketides

**Figure 12:**
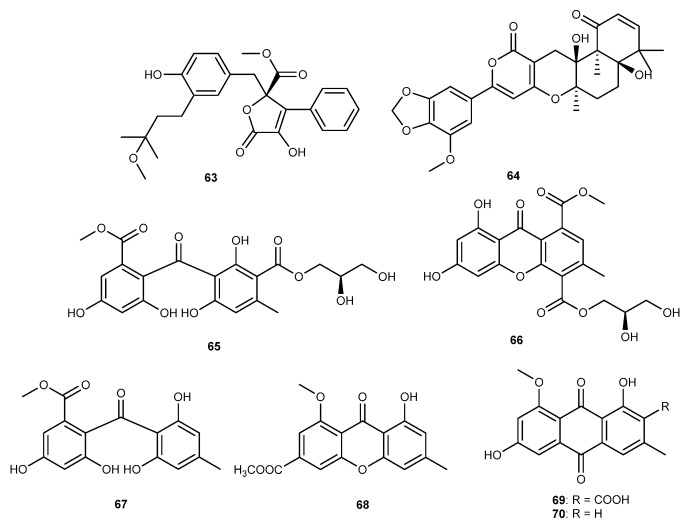
Chemical structures of compounds 63−70.

A detailed chemical investigation of a coral-associated fungus *A. terreus*, cultured from the coral *S. subviride* collected from the coast of Xisha Island in the South China Sea, resulted in the isolation of one unusual metabolite, versicolactone G (63, Figure 12), along with a known analog, territrem A (64, Figure 12) [25]. They were shown to potent anti-inflammatory activity with IC_50_ values of 15.72 and 29.34 μM, respectively, against LPS-induced NO production [25]. *Aspergillus europaeus* WZXY-SX-4-1 was found in the marine sponge *Xestospongia testudinaria*, and produced two new polyketide derivatives, eurobenzophenone B (65, Figure 12), xanthone A (66, Figure 12), along with four known compounds, 3-de-*O*-methylsulochrin (67, Figure 12), yicathin B (68, Figure 12), dermolutein (69, Figure 12), and methylemodin (70, Figure 12) [46]. 3-de-*O*-methylsulochrin showed the significant inhibition against NF-*κ*B pathway in LPS-stimulated SW480 cells [46]. Eurobenzophenone B, xanthone A, yicathin B, dermolutein, and methylemodin showed to inhibit NF-*κ*B pathway and weakly suppressed the expression of NO in LPS-stimulated SW480 cells [46].

**Figure 13:**
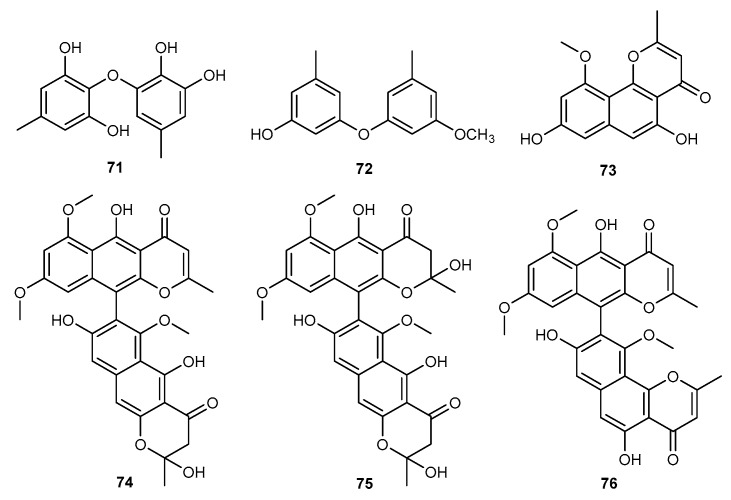
Chemical structures of compounds 71−76.

In order to search for bioactive secondary metabolites from marine fungi, Sen Liu et al. isolated two new metabolites together with six diphenylethers, a diketopiperazine, a chromone, and a xanthone from an EtOAc extract of the fungus *Aspergillus sydowii* J05B-7F-4 associated with the marine sponge *Stelletta* sp. [47]. Among them, only violaceol II (71, Figure 13) and cordyol E (72, Figure 13) displayed weak inhibitory effect against LPS-induced NO production in RAW264.7 cells [47]. A marine fungus, identified as *Aspergillus* sp. SF-6354, was found to produce TMC-256C1 (73, Figure 10) [48]. TMC-256C1 showed considerable anti-neuroinflammatory activity toward the mRNA expression of TNF-*α*, IL-6 and IL-12 production in LPS-activated BV2 cells. This compound also suppressed NO and PGE_2_ production in LPS-activated BV2 cells by the suppression of iNOS and COX-2 protein expression [48]. The surface of a marine algae *Sargassum* sp. from the Yongxing Island, South China Sea, provided *Aspergillus niger* SCSIO Jcsw6F30, which produced three asperpyrone-type bis-naphtho-*γ*-pyrones (BNPs): aurasperone F (74, Figure 13), aurasperone C (75, Figure 13), and asperpyrone A (76, Figure 13) [49]. These compounds possessed significant anti-inflammatory potency through down-regulate the expression of the COX-2 protein in LPS-activated RAW264.7 macrophages with IC_50_ values of 11.1, 4.2, and 6.4 μM, respectively [49].

**Figure 14:**
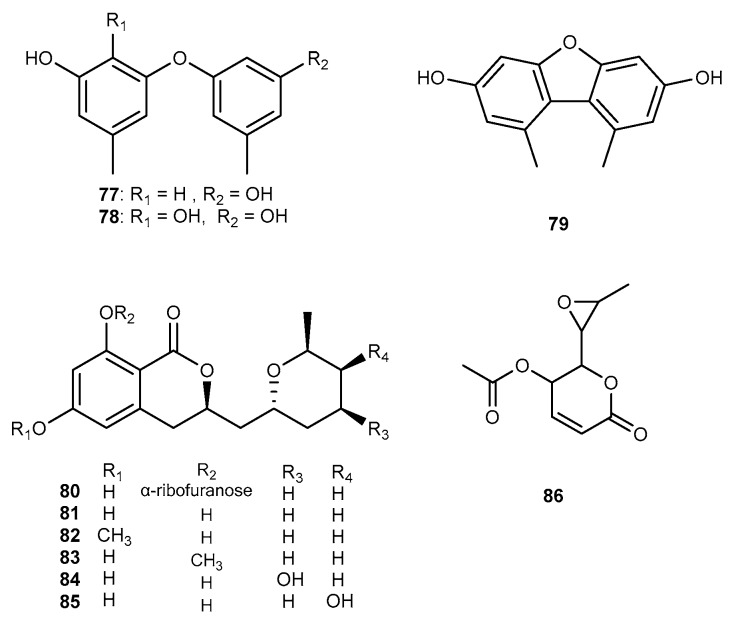
Chemical structures of compounds 77−86.

Two new compounds together with 10 known compounds were detected in the EtOAc extract of the fungal strain *Aspergillus* sp. SCSIO Ind09F01, which was isolated from the deep-sea sediment sample of Indian Ocean [50]. Among them, only three known compounds, diorcinol (77, Figure 14), cordyol C (78, Figure 14), and 3,7-dihydroxy-1,9-dimethyldibenzofuran (79, Figure 14) possessed the inhibitory effects on the expression of COX-2 with the IC_50_ values from 2.4 to 10.6 μM [50]. Dong-Cheol Kim et al. isolated a new dihydroisocoumarin derivative, cladosporin 8-*O*-*α*-ribofuranoside (80, Figure 14), along with five known metabolites, cladosporin (81, Figure 14), asperentin 6-*O*-methyl ether (82, Figure 14), cladosporin 8-*O*-methyl ether (83, Figure 14), 4′-hydroxyasperentin (84, Figure 14), and 5′-hydroxyasperentin (85, Figure 14) from the EtOAc extracts of marine-derived fungus *Aspergillus* sp. SF-5974 and *Aspergillus* sp. SF-5976, obtained from an unidentified red macroalgae collected using a dredge at a depth of 300 m at the Ross Sea [51]. These compounds showed to inhibit the production of NO and PGE_2_ in LPS-stimulated microglial cells with IC_50_ values ranging from 20 to 65 μM due to suppressing the expression of iNOS and COX-2, respectively [51]. Furthermore, cladosporin 8-*O*-*α*-ribofuranoside exhibited the suppression of the phosphorylation and degradation of I*κ*B-*α* and NF-*κ*B, and also reduced the activation of p38 mitogen-activated protein kinase (MAPK) [51]. The marine-derived *Aspergillus* sp. SF-5044 produced a crystalline metabolite, asperlin (86, Figure 14) [52]. The isolated compound 86 was evaluated for its anti-inflammatory potency. It suppressed the expression of the iNOS protein and reduced iNOS-derived NO, inhibited the expression of the COX-2 protein and reduced the COX-derived PGE2 in murine peritoneal macrophages and RAW264.7 caused by activated of LPS [52]. Compound 86 also can reduce the production of pro-inflammatory cytokines including TNF-*α* and IL-1*β*. In addition, it suppressed the phosphorylation of I*κ*B-*α* and the p65 nuclear translocation [52]. Further, compound 86 reduced the expression of pro-inflammatory cytokines and mediators in LPS-activated RAW264.7 cells by increasing HO activity [52].

**Figure 15:**
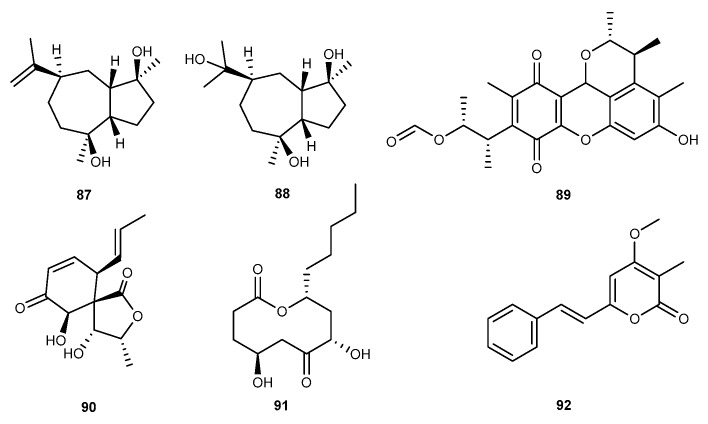
Chemical structures of compounds 87−92.

Guaiadiol A (87, Figure 15) and 4,10,11-trihydroxyguaiane (88, Figure 15) were obtained from marine fungus *P. thomii* KMM 4667 [33]. These compounds exhibited anti-inflammatory effects against NO production in LPS-stimulated murine macrophages by 24.1% ± 2.7%, and 36.6% ± 6.4%, respectively, at the concentration of 10.0 μM [33]. Nguyen Thi Thanh Ngan et al., isolated citrinin H1 (89, Figure 15) from the marine-derived fungal strain *Penicillium* sp. SF-5629 [53]. Citrinin H1 was found to be active on inhibitory effects on the production of NO and PGE_2_ in LPS-activated BV2 microglia, with IC_50_ values of 8.1 ± 1.9 and 8.0 ± 2.8 μM [53]. Penicillospirone (90, Figure 15), a new polyketide-type metabolite, was isolated from an EtOAc extract of the sea-derived fungal *Penicillium* sp. SF-5292 [27]. Penicillospirone exerted the anti-inflammatory effect on iNOS derived NO and COX-2 derived PGE_2_ production with IC_50_ values from 21.9 to 27.6 μM in RAW264.7 macrophages and BV2 microglia stimulated by LPS [27]. Furthermore, penicillospirone also suppressed the mRNA expression of proinflammatory cytokines, including TNF-*α*, IL-1*β*, IL-6, and IL-12. In the further evaluation, penicillospirone was shown to inhibit NF-*κ*B pathway in RAW264.7 and BV2 cells stimulated by LPS [27]. Chemical study was applied to the EtOAc extract of marine *Penicillium* sp. SF-5292, resulting in the discovery of a new 10-membered lactone, penicillinolide A (91, Figure 15) [54]. Penicillinolide A inhibited the NO and PGE_2_ production by suppressing the expression of iNOS and COX-2 in LPS-stimulated macrophages, with IC_50_ values of 20.47 and 17.54 μM [54]. Penicillinolide A also inhibited the mRNA expression of TNF-*α*, IL-1*β,* and IL-6 with IC_50_ values of 8.63, 11.32, and 20.92 μM due to the degradation of I*κ*B-*α*, NF-*κ*B nuclear translocation, and NF-*κ*B DNA binding activity [54]. Dong-Sung Lee et al., successfully purified penstyrylpyrone (92, Figure 15), a styrylpyrone-type metabolite from the methylethylketone extract of sea-derived fungus *Penicillium* sp. JF-55 colonizing in an unidentified sponge gathered from the shores of Jeju Island [55]. Penstyrylpyrone inhibited the overproduction of NO and PGE_2_ with IC_50_ values of 12.32 and 9.35 μM in LPS-stimulated murine peritoneal macrophages and these inhibitory activities were correlated with the overexpressions of iNOS and COX-2, respectively. Penstyrylpyrone also inhibited the mRNA expression of pro-inflammatory cytokines such as TNF-*α*, IL-1*β* with IC_50_ values of 13.54 and18.32 μM [55]. In addition, penstyrylpyrone was shown to inhibit I*κ*B-*α* pathway and the NF-*κ*B DNA-binding activity in LPS-stimulated murine peritoneal macrophages [55].

**Figure 16:**
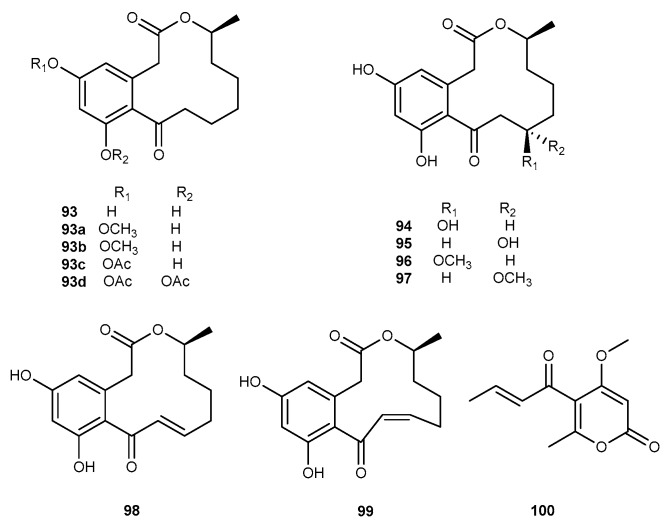
Chemical structures of compounds 93−100.

Chemical study on a marine-derived fungal strain *Penicillium* sp. SF-5859 resulted in the discovery of seven compounds, namely curvularin (93, Figure 16), (11*R*,15*S*)-11-hydroxycurvularin (94, Figure 16), (11*S*,15*S*)-11-hydroxycurvularin (95, Figure 16), (11*R*,15*S*)-11-methoxycurvularin (96, Figure 16), (11*S*,15*S*)-11-methoxycurvularin (97, Figure 16), (10*E*,15*S*)-10,11-dehydrocurvularin (98, Figure 16), and (10*Z*,15*S*)-10,11-dehydrocurvularin (99, Figure 16) [56]. These analogs exhibited strong inhibitory effects on NO and PGE_2_ with IC_50_ values ranging from 1.9 to 18.1 µM, and from 2.8 to 18.7 µM, respectively, in RAW264.7 cells induced by LPS [56]. Compound 99 also suppressed the production of iNOS and COX-2. Furthermore, (10*E*,15*S*)-10,11-dehydrocurvularin exhibited to inhibit the NF-*κ*B pathway [56]. The fungus *Penicillium paxilli* Ma(G)K isolated from a sponge sample *Mycale angulosa*, produced a novel compound pyrenocine A (100, Figure 16), which possessed considerable anti-inflammatory effect against TNF-*α* and PGE_2_ in LPS-stimulated macrophages [57].

**Figure 17:**
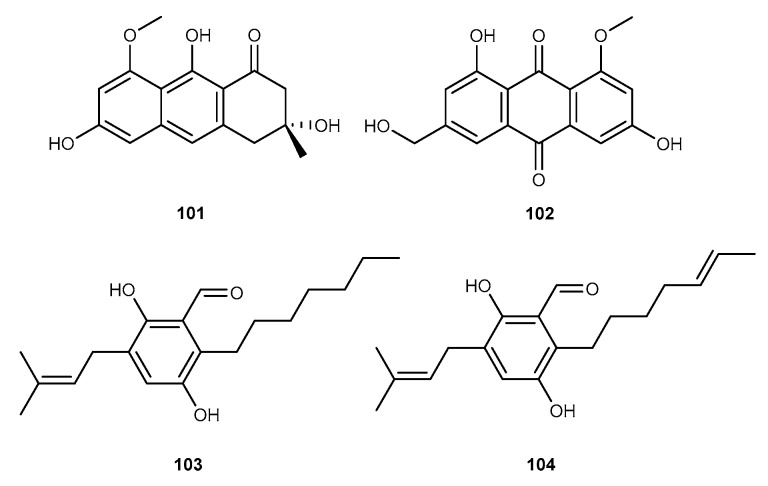
Chemical structures of compounds 101−104.

Asperflavin (101, Figure 17) was isolated from the sea-derived fungus *Eurotium amstelodami* [58]. Asperflavin displayed the overproduction of proinflammatory mediators NO and PGE_2_ in LPS-stimulated RAW264.7 cells by 4.6% and 55.9% at the concentration of 200 μM. Additionally, asperflavin possessed the expression of mRNA proinflammatory cytokines, including TNF-*α*, IL-1*β*, IL-6, and IL-12 [58]. Chemical study of the marine-derived fungus *E. amstelodami* separated from an unidentified marine animal collected from the Sungsan coast in Jeju Island, Korea, have been found an anthraquinone analog, questinol (102, Figure 17) [59]. Questinol showed considerable inhibitory effect on NO and PGE_2_ production in LPS-stimulated RAW264.7 cells with the inhibition rates of 73.0% and 43.5% at the concentrations of 200 μM and also displayed to inhibit the production of pro-inflammatory cytokines such as TNF-*α*, IL-1*β*, and IL-6 [59]. Furthermore, questinol also suppressed the protein expression of iNOS but weak inhibited the protein expression of COX-2 at the concentration of 200 μM [59]. Two benzaldehyde-type fungal analogs, flavoglaucin (103, Figure 17) and isotecrahydro-auroglaucin (104, Figure 17) were extracted in culture extracts of *Eurotium* sp. SF-5989 [60]. Compounds 103 and 104 can suppress the production of pro-inflammatory mediators, NO and PGE_2_, and these inhibitory activities were mediated by inhibiting the expression of COX-2 and iNOS in RAW264.7 macrophages stimulated by LPS. The anti-inflammatory activities of compounds 103 and 104 were due to attenuation of major signaling pathways, NF-*κ*B pathway in LPS-stimulated RAW264.7 macrophages. Furthermore, the anti-inflammatory effects of these were observed through reduction of HO-1 expression regulated by nuclear transcription factorE2-related factor 2 (Nrf2) [60].

**Figure 18:**
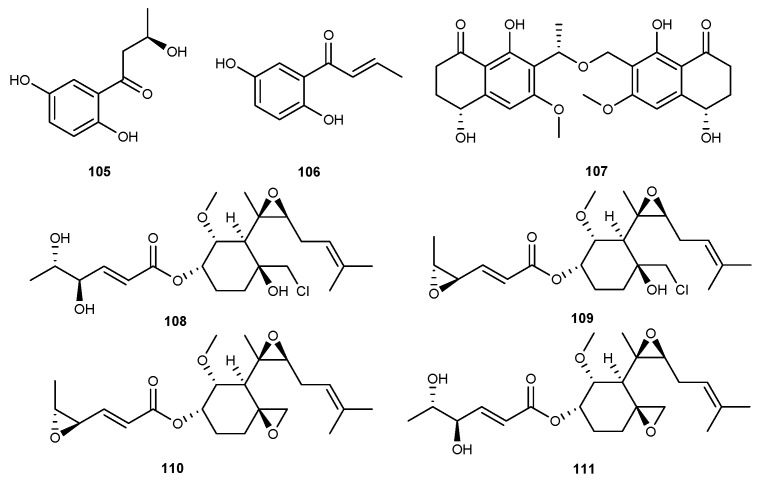
Chemical structures of compounds 105−111.

One new phenolic metabolite, 1-(2,5-dihydroxyphenyl)-3-hydroxybutan-1-one (105, Figure 18), along with 1-(2,5-dihydroxyphenyl)-2-buten-1-one (106, Figure 18), were found in the EtOAc extract of the marine endophytic fungus *Paraconiothyrium* sp. VK-13 [61]. 1-(2,5-dihydroxyphenyl)-3- hydroxybutan-1-one and 1-(2,5-dihydroxyphenyl)-2-buten-1-one displayed inhibitory effect against iNOS derived NO and COX-2 derived PGE_2_ in LPS-stimulated RAW264.7 cells, with IC_50_ values from 3.9 and 12.5 µM [61]. The anti-inflammatory effects of these compounds were attributed to the significant inhibition of the expression of iNOS and COX-2 proteins and the inhibition of mRNA expression of anti-pressure cytokines including TNF-*α*, IL-1*β*, IL-6, and IL-12 [61]. From a marine crinoid collected in Xuwen, Zhanjing City, Guangdong Province, China, fungal strain *Leptosphaerulina chartarum* 3608, was selected for chemical study [62]. One new secondary metabolite, (4*R*,10*S*,4’*S*)-leptothalenone B (107, Figure 18), was discovered in the fungus [62]. It was observed to suppress NO production in LPS-induced RAW264.7 cells, with an IC_50_ value of 44.5 µM [62]. A detailed chemical investigation of an in-house marine-derived fungi *Phoma* sp. NTOU4195 associated with the marine red alga *P. capillacea* resulted in the isolation of three novel polyketides with anti-inflammatory activity, phomaketides A−C (108−110, Figure 18), along with a known analog, FR-111142 (111, Figure 18) [63]. Phomaketides A−C and FR-111142 exhibited strong inhibitory effect on NO production murine macrophage RAW264.7 cells induced by LPS [63]. Phomaketides C exerted the most significant inhibition activity with E_max_ and IC_50_ value of 100% and 8.8 μM, respectively [63].

**Figure 19:**
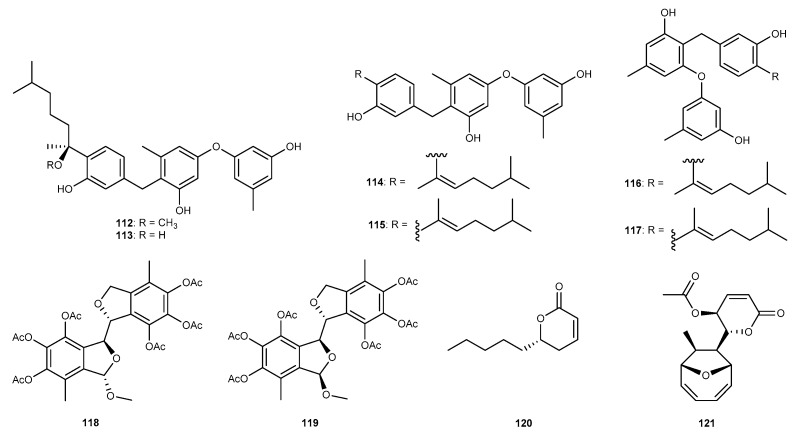
Chemical structures of compounds 112−121.

Expansols A−F (112−117, Figure 19) were polyphenols that were isolated from the marine-derived fungus *Glimastix* sp. ZSDS1-F11 associated with marine sponge samples, *P. fusca* gathered from the Yongxing island of Xisha [64]. All these compounds strongly suppressed the protein expression of COX-2 with IC_50_ values of 3.1, 5.6, 3.0, 5.1, 3.2, and 3.7 µM, respectively [64]. Furthermore, expansols A−F also exhibited strong COX-1 inhibitory activity with IC_50_ values of 5.3, 16.2, 30.2, 41.0 and 56.8 µM, respectively [64]. Two isobenzofuran dimers, spicarins C (118, Figure 19) and D (119, Figure 19), were purified from the marine-derived fungus *Spicaria elegans* KLA03 collected from the marine sediments in Jiaozhou Bay, China [65]. These compounds inhibited the overproduction of NO in BV2 microglial cells induced with LPS with IC_50_ values of 30 and 75 µM, respectively [65]. Chemical study of the sea-derived fungus *Hypocreales* sp. strain HLS-104 isolated from a sponge *G. carnosa* colonizing in the South China Sea afforded two derivatives, (*R*)-5,6-dihydro-6-pentyl-2*H*-pyran-2-one (120, Figure 19) [36]. They showed moderate anti-inflammatory activity against the production of the NO in LPS-treated RAW264.7 cells with average maximum inhibition (E_max_) values of 26.46% at 1 μM [36]. The polyketide mycoepoxydiene (121, Figure 19) was discovered from *Diaporthe* sp. HLY-1, which was isolated from submerged rotten leaves of *Kandelia candel* collected in a mangrove forest in Fujian Province, China [66]. Compound 121 markedly suppressed the LPS-stimulated production of pro-inflammatory mediators and cytokines such as NO, TNF-*α*, IL-6, and IL-1*β* in macrophages [66]. Furthermore, the effect of compound 121 on LPS-stimulated activation were due to block the NF-*κ*B pathway and MAPK signaling pathway [66].

## 5. Peptides

**Figure 20:**
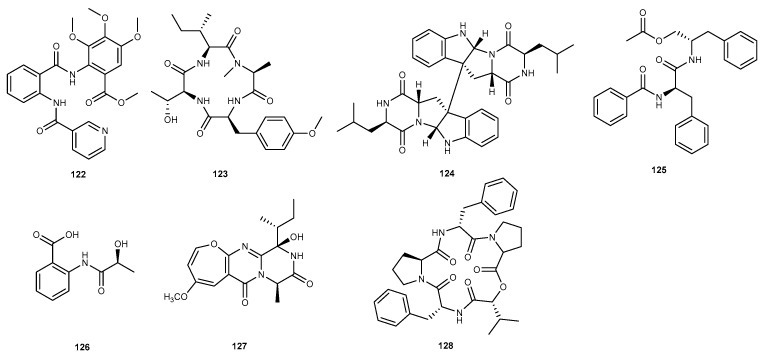
Chemical structures of compounds 122−128.

Methyl 3,4,5-trimethoxy-2-(2-(nicotinamido) benzamido) benzoate (122, Figure 20), was isolated from a coral-associated fungus *A. terreus* associated with the coral *S subviride*, which was gathered from Xisha Island in the South China Sea [25]. This compound showed a considerable anti-inflammatory activity with an IC_50_ value of 5.48 μM [25]. Bioassay-guided investigation of the EtOAc extract of marine sponge-derived fungus *Aspergillus violaceofuscus* afforded new anti-inflammatory activity metabolites named violaceotide A (123, Figure 20) and diketopiperazine dimer (124, Figure 20) [67]. The fungus *A. violaceofuscus* was isolated from the inner part of the marine sponge *Reniochalina* sp. collected from the Xisha Islands in the South China Sea. Violaceotide A and diketopiperazine dimer reduced IL-10 expression in THP-1 cells stimulated by LPS with inhibitory rate of 84.3% and 78.1% at concentration of 10 μM, respectively [67]. Investigation of biologically active peptides from the marine fungus *Aspergillus* sp. SF-5921 (from an unidentified sponge, Sea of Ross) resulted in isolation of aurantiamide acetate (125, Figure 20) [68]. Compound 125 showed inhibitory potency against the LPS-stimulated production of NO and PGE_2_ with IC_50_ values of 49.70 and 51.3 μM in BV2 microglia cells [68]. In addition, it has anti-neuron-flammatory effects through its inhibition of the NF-*κ*B, c-Jun N-terminal kinases (JNK), and p38 pathways [68]. (*S*)-2-(2-hydroxypropanamido) benzoic acid (126, Figure 20), a novel benzoic acid, was isolated as natural product from a sponge-derived marine fungus *P. chrysogenum* SYP-F-2720 [69]. Compound 126 exhibited stronger anti-inflammatory activity than aspirin (swelling rate of 193%) with the swelling rate of 191% in the mouse ear edema model induced by xylene when administered at 100 mg/kg [69]. Chemical investigation of a marine-derived fungus *Acremonium* sp. from the surface of the Caribbean tunicate *Ecteinascidia turbinata*. yielded a new peptide derivative, oxepinamide A (127, Figure 20) [70]. Oxepinamide A showed potent anti-inflammatory effect in a topical resiniferatoxin (RTX)-induced mouse ear edema assay, with the inhibition rate of 82% at the standard testing dose of 50 μg per ear [70]. Alternaramide (128, Figure 20), a marine *Alternaria* sp. SF-5016 metabolite, was interesting in that it contained unusual hydrophobic D-amino acid residues [71]. Compound 128 suppressed the production of PGE_2_ and NO, and these inhibitory effects were correlated with down-regulation of iNOS and COX-2 expression in LPS-induced RAW264.7 and BV2 macroglia cells with IC_50_ values ranging from 27.63 to 40.52 μM [71]. It also inhibited pro-inflammatory cytokines, such as TNF-*α*, IL-1*β*, IL-6, and IL-12 in LPS-induced RAW264.7 and BV2 macroglia cells. In addition, the compound 128 suppressed the NF-*κ*B and MAPK signaling pathway. Furthermore, compound 128 significantly reduced the Toll-like receptor 4 (TLR4) and myeloid differentiation primary response gene 88 (MyD88) in LPS-induced RAW264.7 and BV2 macroglia cells at the mRNA and protein levels [71]. 

## 6. Others

**Figure 21:**
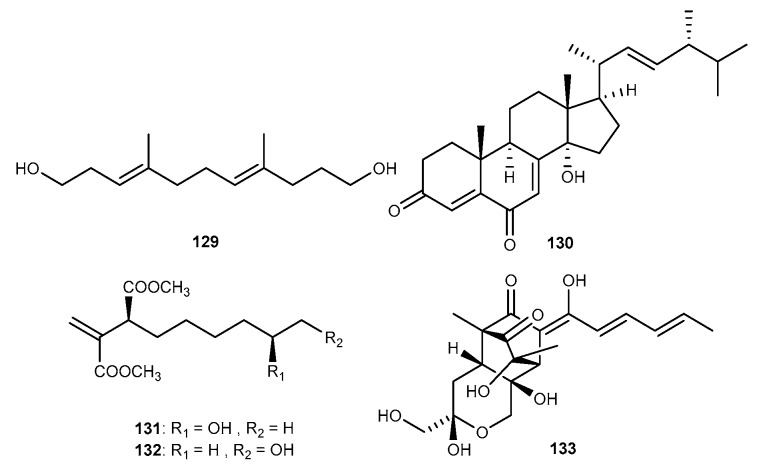
Chemical structures of compounds 129−133.

A novel linear aliphatic alcohol, (3*E*,7*E*)-4,8-di-methyl-undecane-3,7-diene-1,11-diol (129, Figure 21), together with three known compounds, 14*α*-hydroxyergosta-4,7,22-triene-3,6-dione (130, Figure 21) were purified from the coral-associated fungus *A. terreus* associated with the coral *S. subviride*, which was collected from the coast of Xisha Island in the South China Sea [25]. These compounds exhibited considerable inhibitory activity against NO production with IC_50_ values ranging from 17.45 to 29.34 μM [25]. Two hexylitaconic acid derivatives, methyl8-hydroxy- 3-methoxycarbonyl-2-methylenenonanoate (131, Figure 21) and (3*S*)-methyl9-hydroxy-3- methoxycarbonyl-2-methylenenonanoate (132, Figure 21), were separated from the EtOAc extract of the fungal strain *Penicillium* sp. (J05B-3-F-1) colonizing in a sponge *Stelletta* sp. collected from the coast of Jeju island, Korea [72]. The two isolates weakly inhibited the production of IL-1*β* at the concentration of 200 μM [72]. Trichodermanone C (133, Figure 21) was isolated from the marine fungal strain A12 of *Trichoderma citrinoviride* associated with the green alga *Cladophora* sp. collected in Italy [73]. Trichodermanone C was evaluated to show strong inhibitory effect on nitrite levels in LPS-stimulated J774A.1 macrophages [73].

## 7. Conclusions

The inflammatory disease is one of the most common diseases around the world [74]. Recent literatures showed that the prevalence, severity, and complexity of the disease were rising rapidly and adding to the healthcare costs considerably [75]. What is more, the inflammatory disease operates by an advanced system and has a broad influence on physiological aspects and human pathology. Currently, with the development of the synthetic drug formulation, some classes of anti-inflammatory drugs such as aspirin, nonsteroidal anti-inflammatory drugs (NSAIDs), and corticosteroids are used in the clinic [76]. However, all the therapeutics can cause quite harmful side effects to human beings after long-term and high-dose medication. Marine fungi have the potential ability to produce diverse chemical structures with anti-inflammatory activities. During 2000–2018, about 133 anti-inflammatory compounds in 52 references belonging to five diverse chemical classes were reported, including alkaloids, terpenoids, polyketides, peptides, and others. Over 50 compounds were found to display significantly anti-inflammatory activities. For example, preussions G (5) and I (7), graphostromanes F (28), khusinol B (29), and mangicol A (31), which IC_50_ values or reduction were even stronger than that positive control. From distribution point of view, 75% of all anti-inflammatory structures were polyketides and terpenoids indicating that polyketides and terpenoids have great potential in the development of anti-inflammatory drugs. This review provided a lot of potential lead compounds for finding novel anti-inflammatory agents from marine-derived fungi, especially, *Aspergillus* (41.4%), and *Penicillium* (27.1%). Lots of potential agents derived from marine fungi were found to have significant effects against inflammation. Therefore, it could be suggested that marine fungi-derived natural products will play a vital role in developing novel drugs against inflammation with satisfactory tolerability for long-term use [77,78].

## Figures and Tables

**Table 1:** Anti-inflammatory alkaloids from marine fungi.

Metabolites	Species	Activities	Reference
Preussins C–K (1–9)	*A. flocculosus* 16D-1	against IL–6 with IC_50_ values of 0.11–22 μM in LPS-activated THP-1	[23]
Asperversiamides B, C, F, G (10–13)	*A. versicolor*	against iNOS with IC_50_ values of 5.39–16.58 μM in LPS-activated RAW264.7 cells	[24]
Luteoride E (14)	*A. terreus*	against NO with IC_50_ value of 24.65 μM in LPS-activated RAW264.7 cells	[25]
Chrysamide C (15)	*P. chrysogenum* SCSIO41001	against IL–6 with 40.06% inhibitory at 1.0 μM	[26]
Viridicaol (16)	*Penicillium* sp. SF-5295	against NO and PGE_2_ with IC_50_ values of 46.03 and 30.37 μM in LPS-activated RAW264.7 and 43.03 and 34.20 μM in LPS-activated BV2 cells	[27]
Brevicompanines E, H (17, 18)	*Penicillium* sp.	against NO with IC_50_ values of 27 and 45 μg/mL in LPS-activated RAW264.7 cells	[28]
Methylpenicinoline (19)	*Penicillin* sp. SF-5995	against NO, PGE2, iNOS, and COX-2 with IC_50_ values from 34 to 49 μM	[29]
Neocechinulin A (20)	*Eurotium* sp. SF-5989	significantly affection at concentrations exceeding 25 µM	[30]

**Table 2:** Anti-inflammatory terpenoids from marine fungi.

Metabolites	Species	Activities	Reference
Brasilanones A and E (21, 22)	*A. terreus* CFCC 81836	against NO with 47.7% and 37.3% inhibition rates at 40 μM in LPS-activated RAW264.7 cells	[31]
Dihydrobipolaroxins B−D (23−25) Dihydrobipolaroxin (26)	*Aspergillus* sp. SCSIOW2	against NO with moderate anti-inflammatory effects	[32]
Thomimarine E (27)	*P. thomii* KMM 4667	against NO with 22.5% inhibition rate at 10.0 μM in LPS-activated RAW264.7 cells	[33]
Graphostromane F (28)	*Graphostroma* sp. MCCC 3A00421	against NO with IC_50_ value of 14.2 μM in LPS-activated RAW264.7 cells	[34]
Khusinol B (29)	*Graphostroma* sp. MCCC 3A00421	against NO with IC_50_ values of 17 μM in LPS-activated RAW264.7 cells	[35]
1*R*,6*R*,7*R*,10*S*-10-hydroxy-4(5)-cadinen-3-one (30)	*Hypocreales* sp. HLS-104	against NO with E_max_ values of 10.22% at 1 μM in LPS-activated RAW264.7 cells	[36]
Mangicols A and B (31, 32)	*F. heterosporum* CNC-477	81% and 57% inhibition rate at 50 μg per ear in PMA-induced mouse ear edema assay	[37]
Chondroterpenes A, B, H (33–35) Hirsutanol A (36) Chondrosterins A, B (37, 38)	*Chondrostereum* sp. NTOU4196	against NO with considerable inhibitory effects at 20 μM in LPS-activated BV-2 cells	[38]
Lovastatin (39)	*A. terreus*	against NO with IC_50_ value of 17.45 μM in LPS-activated RAW264.7 cells	[25]
Aspertetranones A−D (40−43)	*Aspergillus* sp. ZL0-1b14	against IL-6 with 43% and 69% inhibition rates at 40 μM in LPS-activated RAW264.7 cells	[39]
Pleosporallins A−C (44−46)	*Phoma* sp. NTOU4195	against IL-6 with about 30.0% inhibition rate at 5–20 μg/mL in LPS-activated RAW264.7 cells	[40]
7-acetoxydehydroaustinol (47) Austinolide (48) 7-acetoxydehydroaustin (49) 11-hydroxyisoaustinone (50) 11-acetoxyisoaustinone (51)	*Penicillium* sp. SF-5497	against NO with IC_50_ values of 61.0, 30.1, 58.3, 37.6, and 40.2 μM in LPS-activated BV-2 cells	[41]
Citreohybridonol (52)	*P. atrovenetum*	anti-neuroinflammatory activity	[42]
Tanzawaic acid Q (53) Tanzawaic acids A (54), C (55), D (56), and K (57)	*P. steckii* 108YD142	against NO with considerably anti-inflammatory activity in LPS-activated RAW264.7 cells	[43]
2*E*,4*Z*-tanzawaic acid D (58) Tanzawaicacids A (54), E (59)	*Penicillium* sp. SF-6013	against NO with IC_50_ values of 37.8, 7.1, and 42.5 μM in LPS-activated RAW264.7 cells	[44]
Stachybotrysin C (60), Stachybonoid F (61), Stachybotylactone (62)	*S. chartarum* 952	against NO with IC_50_ values of 27.2, 52.5, and 17.9 μM in LPS-activated RAW264.7 cells	[45]

**Table 3:** Anti-inflammatory polyketides from marine fungi.

Metabolites	Species	Activities	Reference
Versicolactone G (63) Territrem A (64)	*A. terreus*	against NO with IC_50_ values of 15.72 and 29.34 μM in LPS-activated RAW264.7 cells	[25]
Eurobenzophenone B (65) Canthone A (66) 3-de-*O*-methylsulochrin (67) Yicathin B (68) Dermolutein (69) Methylemodin (70)	*A. europaeus* WZXY-SX-4-1	66 against NF-*κ*B with significant inhibition in LPS-activated SW480 cells 65, 67, 68, 69, 70 against NF-*κ*B with inhibition and against NO with weak inhibition in LPS-activated SW480 cells	[46]
Violaceol II (71) Cordyol E (72)	*A. sydowii* J05B-7F-4	against NO with weak inhibition in LPS-activated RAW264.7 cells	[47]
TMC-256C1 (73)	*Aspergillus* sp. SF-6354	against NO and PGE_2_ with considerable anti-neuroinflammatory activity in LPS-activated BV2 cells	[48]
Aurasperone F (74) Aurasperone C (75) Asperpyrone A (76)	*A. niger* SCSIO Jcsw6F30	against COX-2 with IC_50_ values of 11.1, 4.2, and 6.4 μM in LPS-activated RAW264.7 cells	[49]
Diorcinol (77) Cordyol C (78) 3,7-dihydroxy-1,9-Dimethyldibenzofuran (79)	*Aspergillus* sp. SCSIO Ind09F01	against the COX-2 expression with IC_50_ values of 2.4−10.6 μM	[50]
Cladosporin 8-*O*-*α*-ribofuranoside (80) Cladosporin (81) Asperentin 6-*O*-methyl ether (82) Cladosporin 8-*O*-methyl ether (83) 4′-hydroxyasperentin (84) 5′-hydroxyasperentin (85)	*Aspergillus* sp. SF-5974 and *Aspergillus* sp. SF-5976	against NO and PGE_2_ with IC_50_ values of 20−65 μM in LPS-activated microglial cells	[51]
Asperlin (86)	*Aspergillus* sp. SF-5044	against NO and PGE_2_ in LPS-activated murine macrophages	[52]
Guaiadiol A (87) 4,10,11-trihydroxyguaiane (88)	*P. thomii* KMM 4667	against NO with 24.1% and 36.6% inhibition at 10.0 μM in LPS-activated murine macrophages	[33]
Citrinin H1 (89)	*Penicillium* sp. SF-5629	against NO with IC_50_ values of 8.1 and 8.0 μM in LPS-activated BV2 cells	[53]
Penicillospirone (90)	*Penicillium* sp. SF-5292	against NO and PGE_2_ with IC_50_ values of 21.9–27.6 μM in LPS-activated RAW264.7 and BV2 cells	[27]
Penicillinolide A (91)	*Penicillium* sp. SF-5292	against NO, PGE_2_, TNF-α, IL-1β and IL-6 with IC_50_ values of 20.47, 17.54, 8.63, 11.32, and 20.92 μM in LPS-activated RAW264.7 and BV2 cells	[54]
Penstyrylpyrone (92)	*Penicillium* sp. JF-55	against NO, PGE_2_, TNF-α, IL-1β with IC_50_ values of 12.32, 9.35, 13.54, and 18.32 μM in LPS-activated murine peritoneal macrophages	[55]
Curvularin (93), (11*R*,15*S*)-11-hydroxycurvularin (94) (11*S*,15*S*)-11-hydroxycurvularin (95) (11*R*,15*S*)-11-methoxycurvularin (96) (11*S*,15*S*)-11-methoxycurvularin (97) (10*E*,15*S*)-10,11-dehydrocurvularin (98) (10*Z*,15*S*)-10,11-dehydrocurvularin (99)	*Penicillium* sp. SF-5859	against NO and PGE_2_ with IC_50_ values of 1.9–18.1, and 2.8–18.7 µM in LPS-activated RAW264.7 cells	[56]
Pyrenocine A (100)	*P. paxilli*	against TNF-α and PGE_2_ in LPS-activated macrophages	[57]
Asperflavin (101)	*E. amstelodami*	against NO and PGE_2_ with 4.6% and 55.9% inhibition rates to NO and PGE_2_ at 200 μM in LPS-activated RAW264.7 cells	[58]
Questinol (102)	*E. amstelodami*	against NO and PGE_2_ with 73.0% and 43.5% inhibition rates at 200 μM against NO and PGE_2_	[59]
Flavoglaucin (103) Isotecrahydro-auroglaucin (104)	*Eurotium* sp. SF-5989	against NO and PGE_2_ in LPS-activated RAW264.7 cells	[60]
1-(2,5-dihydroxyphenyl)-3-hydroxybutan-1-one (105) 1-(2,5-dihydroxyphenyl)-2-buten-1-one (106)	*Paraconiothyrium* sp. VK-13	against NO and PGE_2_ with IC_50_ values of 3.9–12.5 µM in LPS-activated RAW264.7 cells	[61]
(4*R*,10*S*,4’*S*)-leptothalenone B (107)	*L. chartarum* 3608	against NO with IC_50_ value of 44.5 µM in LPS-activated RAW264.7 cells	[62]
Phomaketides A−C (108−110) FR-111142 (111)	*Phoma* sp. NTOU4195	against NO with E max and IC_50_ value of 100% and 8.8 μM in LPS-activated RAW264.7 cells	[63]
Expansols A−F (112−117)	*Glimastix* sp. ZSDS1-F11	against expression of COX-2 with IC_50_ values of 3.1, 5.6, 3.0, 5.1, 3.2, and 3.7 µM against expression of COX-1 with 5.3, 16.2, 30.2, 41.0, and 56.8 µM	[64]
Spicarins C (118) and D (119)	*S. elegans* KLA03	against NO with IC_50_ values of 30 and 75 µM in LPS-activated BV2 cells	[65]
(*R*)-5,6-dihydro-6-pentyl-2*H*-pyran-2-one (120)	*Hypocreales* sp. strain HLS-104	against NO with E_max_ value of 26.46% at 1 μM in LPS-activated RAW264.7 cells	[36]
Mycoepoxydiene (121)	*Diaporthe* sp. HLY-1	against NO and TNF-α, IL-6, and IL-1β in LPS-activated macrophages	[66]

**Table 4:** Anti-inflammatory peptides from marine fungi.

Metabolites	Species	Activities	Reference
Methyl 3,4,5-trimethoxy-2-(2-(nicotinamido)benzamido)benzoate (122)	*A. terreus*	against NO with IC_50_ value of 5.48 μM in LPS-activated RAW264.7 cells	[25]
Violaceotide A (123) Diketopiperazine dimer (124)	*A. violaceofuscus*	against IL-10 expression with inhibitory rate of 84.3% and 78.1% at 10 μM in LPS-activated THP-1 cells	[67]
Aurantiamide acetate (125)	*Aspergillus* sp.	against NO and PGE_2_ with IC_50_ values of 49.70 and 51.3 μM in LPS-activated BV2 cells	[68]
(*S*)-2-(2-hydroxypropanamido) Benzoic Acid (126)	*P. citrinum* SYP-F-2720	with the swelling rate of 191% at 100 mg/kg	[69]
Oxepinamide A (127)	*Acremonium* sp.	inhibition rate of 82% at 50 μg per ear in RTX-activated mouse ear edema assay	[70]
Alternaramide (128)	*Alternaria* sp. SF-5016	against NO and PGE_2_ with IC_50_ values ranging from 27.63 to 40.52 μM in LPS-activated RAW264.7 and BV2 cells	[71]

**Table 5:** Anti-inflammatory other compounds from marine fungi.

Metabolites	Species	Activities	Reference
(3*E*,7*E*)-4,8-di-methyl-undecane-3,7-diene-1,11-diol (129) 14*α*-hydroxyergosta-4,7,22-triene-3,6-dione (130)	*A. terreus*	against NO with IC_50_ values of 17.45 and 29.34 μM in LPS-activated RAW264.7 cells	[25]
Methyl 8–hydroxy–3-methoxycarbonyl-2-methylenenonanoate (131) (3*S*)-Methyl 9-hydroxy-3-methoxycarbonyl-2-methylenenonanoate (132)	*Penicillium* sp. (J05B-3-F-1)	against IL-1*β* with weakly inhibition at 200 μM	[72]
Trichodermanone C (133)	*T. citrinoviride*	strong inhibitory effect on nitrite levels in LPS-activated J774A.1 macrophages	[73]
